# Supplementary material for: Identification of the Antigens Recognised by Colorectal Cancer Patients Using Sera from Patients Who Exhibit a Crohn’s-like Lymphoid Reaction
Source: Biomolecules. 2022 Jul 29;12(8):1058. doi: 10.3390/biom12081058 (PMC9406176; doi:10.3390/biom12081058)
Supplement: Supplementary file 1 [file biomolecules-12-01058-s001.zip › biomolecules-1800687-supplementary.pdf]

Supplementary Materials: Table S1: Patient information for samples analysed by immunohistochemistry (DOI: 10.5281/zenodo.6660264); Table S2: Differential expression of SEREX-defined antigens in colorectal cancer when comparing clinical features using the GSE5206 dataset (DOI: 10.5281/zenodo.5535023), Table S3: Differentially expressed antigens that showed >two-fold difference in expression between (A) AJCC stage (B) T stage (C) collection sites and (D) recurrence type. *p*-values shown (DOI: 10.5281/zenodo.5535019).
